# An integrated immunopathological model of syphilis serofast: a systematic review and meta-analysis

**DOI:** 10.3389/fimmu.2026.1758075

**Published:** 2026-03-30

**Authors:** Yong-Xin Zeng, Lin Yang, Yuan-Yuan He, Zi-Liang Deng, Wu-Jian Ke, Hai-Ying Wang

**Affiliations:** 1 Department of Epidemiology, School of Public Health, Southern Medical University, Guangzhou, Guangdong, China; 2 Hospital for Skin Diseases, Institute of Dermatology, Chinese Academy of Medical Sciences and Peking Union Medical College, Nanjing, Jiangsu, China; 3 National Center for Sexually Transmitted Disease Control, Chinese Center for Disease Control and Prevention, Nanjing, Jiangsu, China; 4 School of Public Health, Nanjing Medical University, Nanjing, Jiangsu, China; 5 Key Laboratory of Infectious Diseases Research in South China (Southern Medical University), Ministry of Education, Guangzhou, Guangdong, China

**Keywords:** cytokines, immunity, meta-analysis, serofast, syphilis, T-lymphocytes

## Abstract

**Objective:**

The immunological mechanisms underlying the syphilis serofast remain incompletely elucidated. This systematic review and meta-analysis aims to quantify the association between key immune indicators and serofast.

**Methods:**

We systematically searched PubMed, Embase, Web of Science, Google Scholar and Chinese databases (CNKI, VIP, and CBMdisc) until December 31, 2024, for case-control, cross-sectional or cohort studies meeting serofast criteria (RPR/TRUST titer ≤1:8 persisting for ≥12 months). Random-effects models were used to calculate standardized mean differences (SMD) with 95% confidence intervals (CIs). The risk of bias was assessed using the Newcastle-Ottawa Scale (for observational studies) by two independent reviewers.

**Results:**

A total of 38 studies involving 5082 patients were included. The serofast group exhibited significant immune dysregulation: (1) Cellular immune suppression:decreased CD4+ T cells (SMD=-0.61, I²=33.5%) and increased CD8+ T cells (SMD = 0.40, I²=66.7%), leading to an inverted CD4+/CD8+ ratio (SMD=-0.44, I²=64.7%); (2) Th1/Th2 shift:suppression of Th1 cytokines (e.g., IFN-γ, SMD=-2.19, I²=95.7%) with a predominant Th2 response (e.g., IL-10, SMD =+ 2.63,I²=92.5%); (3) Humoral abnormalities: persistently elevated IgM (SMD = 0.96,I²=94.4%) and complement consumption (C3,SMD=-0.60, I²=89.0%; C4, SMD=-0.80, I²=87.6%); (4) Signaling dysregulation: downregulated TLR2 expression and disordered chemokine receptors (TLR2 mRNA,SMD=-1.52, I²=36.0%).The substantial heterogeneity (I² > 50%) observed in several analyses was explored in subgroup and sensitivity analyses, as detailed in the main text.

**Conclusions:**

The serofast is characterized by a cascade of “cellular immune suppression-Th1/Th2 shift-complement exhaustion.” Our findings establish a quantified immunological basis for the serofast state and suggest potential targets for immunomodulatory therapy.

**Systematic review registration:**

https://www.crd.york.ac.uk/prospero/, identifier CRD420251156478.

## 1 Introduction

Syphilis, caused by Treponema pallidum(TP), remains a significant global public health threat (1). While standard therapy leads to seroconversion in most patients, a substantial proportion (3.8%-41.0% across stages) (2–4) develop a serofast state, defined as persistent non-treponemal antibody titers (e.g., RPR/TRUST ≤1:8 for ≥12 months) despite adequate treatment. This condition necessitates long-term monitoring, consumes considerable resources, and imposes a psychological burden on patients, highlighting the urgent need to elucidate its underlying mechanisms (5, 6).

Emerging evidence suggests that serofast is intrinsically linked to host immune dysregulation (7–10). A central hypothesis points to a profound imbalance in adaptive immunity, particularly T-lymphocyte subsets, characterized by cellular suppression (e.g., reduced CD4+ T cells) and a skewed Th1/Th2 cytokine profile (diminished IFN-γ, IL-2; elevated IL-10) (11–15). Furthermore, broader immune perturbations have been observed, encompassing aberrant innate immune recognition (e.g., via TLR2 downregulation), humoral abnormalities such as persistent IgM elevation, and complement consumption (16–24).

Despite these insights, the immunological landscape of serofast remains fragmented and poorly quantified. Critically, no prior systematic review or meta-analysis has attempted to quantitatively synthesize this evidence, leaving the field with inconsistent and non-comparable findings across studies. To address this definitive knowledge gap, we conducted this systematic review and meta-analysis to: (1) quantitatively synthesize the evidence linking key immune markers to serofast, and (2) construct an evidence-based pathophysiological model to inform future immunomodulatory therapeutic strategies.

This study was conducted in accordance with PRISMA guidelines (25), and its protocol was prospectively registered on the PROSPERO platform (CRD420251156478).

## 2 Methods

### 2.1 Data sources and search strategy

**Figure 1:**
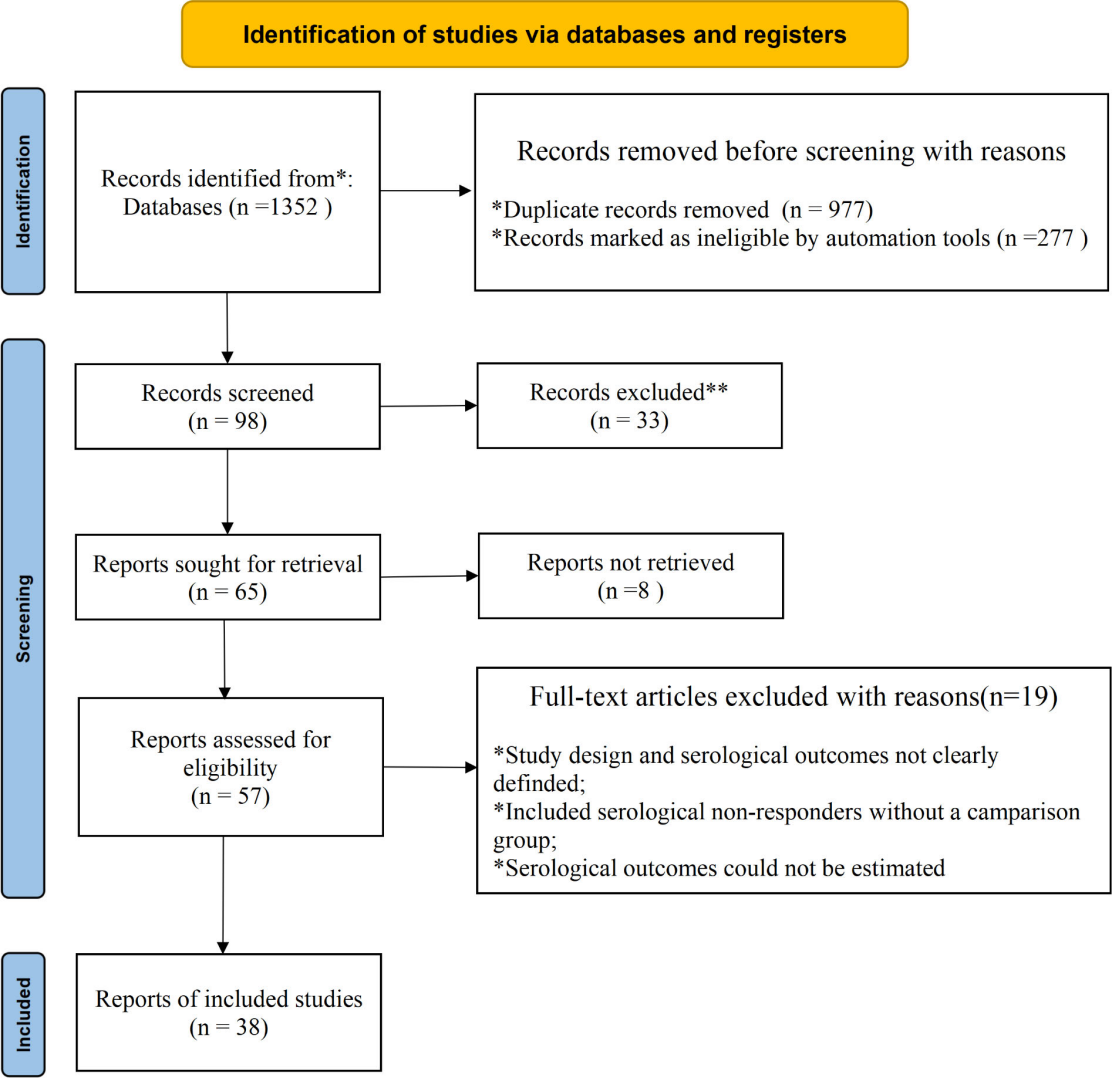
PRISMA flow diagram of the systematic review process using the terms “syphilis” and “serology” and “resistance”, “response”, “serofast”, or “seroresistance” in seven databases.

We systematically searched PubMed, Embase, Web of Science, Google Scholar and Chinese databases (CNKI, VIP, and CBMdisc) until December 31, 2024, for case-control, cross-sectional or cohort studies meeting serofast criteria (RPR/TRUST titer ≤1:8 persisting for ≥12 months). The search strategy, developed based on the PICO framework (Population: syphilis serofast patients; Indicator/Comparison: immune indicators in serofast vs. control groups; Outcome: association with serofast state), combined MeSH terms and free-text keywords for syphilis, serofast, and immune factors (full strategy in **Supplementary Material S1**). Searches across these 7 databases yielded 1,352 records before deduplication, with the detailed contribution of each source presented in the PRISMA flow diagram (**Figure 1**). Reference lists of relevant articles were manually screened. No language or date restrictions were applied initially, but only English or Chinese publications were considered for full-text review due to practical constraints.

### 2.2 Inclusion and exclusion criteria

Inclusion criteria: (1) Studies with a confirmed diagnosis of syphilis serofast (persistent low-titer RPR/TRUST positivity ≥12 months after standardized treatment); (2) Primary focus on the relationship between syphilis serofast and immunological factors; (3) Inclusion of immunological indicator data (e.g., T-cell subsets, cytokines, NK cell activity); (4) Control group comprising serologically cured individuals or healthy subjects; (5) Study design as case-control, cohort, or cross-sectional research; (6) Publications in Chinese or English (due to practical constraints in obtaining and translating full texts in other languages). (7) Immunological assay methods must be clearly defined and traceable.

Cross-sectional studies were included as they can provide valuable snapshot data on immune status, which is relevant for establishing associations, though they preclude causal inference.

Exclusion criteria: (1) Incomplete data or inability to obtain full text; (2) Duplicate publications or non-original research (e.g., reviews, commentaries); (3) Patients with concurrent HIV infection, pregnancy, or lactation; (4) Absence of a control group; (5) Animal studies or *in vitro* research; (6) Patients with concomitant autoimmune diseases or long-term immunosuppressive therapy.

Serological cure criteria: RPR/TRUST seroconversion to negative, or ≥4-fold (2 dilutions) reduction in titer post-treatment compared to baseline.

### 2.3 Data extraction and quality assessment

Two independent reviewers independently screened all retrieved records by title/abstract and subsequently by full text against the predefined eligibility criteria, using a standardized Excel form for the screening process. Any discrepancies were resolved through consensus or by arbitration from a senior researcher. Data were extracted onto a standardized form. The extracted content included: (1) Study basic information: first author, publication year, study design type, study location. (2) Study subject characteristics: total sample size, sample size for serofast group/serum cured group/healthy control group, definition criteria for serofast (e.g., specific duration of persistently low RPR/TRUST titers ≥12 months), definition criteria for serum cure. (3) Outcome indicator data: Mean, standard deviation (SD), and sample size (n) of immune markers across groups. (4) Additional information: Adjusted confounding factors, conclusions, etc. When critical data were missing or unclear, the original authors were contacted via email to request the information. In the data extraction process for this study, while some granular data (e.g., specific subgroup breakdowns) were occasionally missing, the key summary statistics required for our meta-analyses were consistently available from the published results of the included studies. Therefore, the specific step of contacting authors was not undertaken. To ensure robustness, we implemented rigorous measures including dual independent data extraction and sensitivity analyses, which confirmed the stability of our findings.

The methodological quality of included studies was assessed using appropriate tools. For cross-sectional studies, the 11-item Agency for Healthcare Research was employed (26). For cohort and case-control studies, the Newcastle-Ottawa Scale (NOS) was employed. Results are summarized in **Table 1**.

**Table 1:** Study design and characteristics of the study population among 38 studies included in the systematic review.

First author (year)	Location	Study design	Total sample size (n)	Serofast cases (n)	Study period	Risk of bias	Indicators reported
Wei-Chen YAN,2024	Hainan	R cohort	399	202	2017/1-2022/12	High	SMD
Jia-Ping Zhou,2024	Zhejiang	Case-control	102	51	2019/1—2021/6	top	SMD
J.Wang,2024	Zhejiang	Case-control	90	30	2020/1-2022/12	Low	SMD
Y.Zhang,2024	Sichuan	Case-control	250	64	2021/2-2022/4	Low	SMD
G.Wu,2023	Shanghai	Case-control	156	52	2019/6-2021/6	Low	SMD
P.Zhao,2023	Jiangsu	Case-control	131	37	2020/11-2022/11	Low	SMD
Ai-Hong Gong,2021	Shandong	P cohort	107	48	2019/5-2020/11	top	SMD
C.Xu,2021	Zhejiang	P cohort	130	30	–	top	SMD
Xue-Mei Han,2021	Tianjin	Case-control	120	40	2018/3-2020/3	Low	SMD
Y.Guan,2021	Guangong	P cohort	90	30	2014/6-2016/6	Low	SMD
Zhang,2021	Jiangsu	Case-control	90	37	2019/1-2020/5	top	SMD
J.Wen,2021	Shandong	P cohort	120	30	2012/6-2016/10	top	SMD
Dong-Ping Lu,2021	Guangdong	P cohort	100	50	2018/9-2020/9	top	SMD
R.Xiao,2021	Hubei	R cohort	160	80	2017/1-2019/12	top	SMD
R.Zhang,2021	Guangdong	P cohort	120	30	2017/2-2018/6	top	SMD
D.Qiang,2020	Hubei	Case-control	50	20	2016/4-2019/4	top	SMD
J.Xu,2019	Heilongjiang	Case-control	116	58	2015/12-2019	top	SMD
Y.Huang,2018	Shandong	Case-control	206	103	2013/12-2016/12	top	SMD
X.Chen,2018	Ningxia	Case-control	60	30	2015/3-2017/1	top	SMD
Y.Li,2018	Henan	Case-control	160	80	2016/5-2017/5	top	SMD
P.Lin,2016	Zhejiang	Case-control	100	35	2012/10-2014/9	top	SMD
C.Xu,2016	Sichuan	Case-control	105	35	2010/1-2016/1	top	SMD
J.Yuan,2016	Hainan	Case-control	92	19	2014/4-2016/4	top	SMD
Shi-Zhuang Lin,2016	Zhejiang	Case-control	110	35	2010/5-2013/8	top	SMD
Y.Zhou,2015	Hubei	P cohort	60	30	2013/12-2014/12	Low	SMD
Z.Liu,2015	Guangdong	R cohort	115	35	2009/1-2013/1	top	SMD
C.Xu,2015	Zhejiang	R cohort	140	38	–	top	SMD
Xian-Xiang Huang,2014	Sichuan	R cohort	149	38	–	top	SMD
G.Xu,2014	Guangdong	Case-control	540	180	2010/1-2013/1	top	SMD
Yan-Jia Lin,2013	Guangdong	Case-control	325	112	–	top	SMD
Bai-Ling Liu,2013	Jiangsu	P cohort	60	30	–	top	SMD
Hai-Li Liu,2013	Guangxi	Case-control	79	32	2007-2011	top	SMD
Zhao,2012	Jiangsu	Case-control	49	26	–	top	SMD
Dong-Ping Lu,2012	Guangdong	Case-control	100	40	2008/9—2010/9	top	SMD
X,Lin,2010	Jiangsu	Case-control	79	23	2008/7-2009/6	top	SMD
O.Liu,2007	Jiangxi	R cohort	60	20	2004/9-2006/9	top	SMD
Tu-Ya Bao,2006	Guangdong	Case-control	60	38	–	High	SMD
Ri-Dong Yang,2005	Guangdong	Cross-sectional	102	32	–	top	SMD

R, retrospective; P, prospective.

“ - “ indicates that the time period for selecting research subjects is not specified in the article.

Among the 38 included studies, cross-sectional studies were evaluated using the AHRQ scale, while case-control/cohort studies were assessed using the NOS.

### 2.4 Statistical analysis

All analyses were performed using R software (version 4.3.2). Pooled effect estimates were calculated with the meta, presenting risk ratios (RR) for dichotomous outcomes and standardized mean differences (SMD) for continuous outcomes, both with 95% confidence intervals (CIs). Heterogeneity was quantified using the I² statistic and Cochran’s Q test. A random-effects model (between-study variance τ² estimated by restricted maximum likelihood) was applied when substantial heterogeneity was present (I² > 50% or P _Q_< 0.1).

Planned subgroup analyses (e.g., by study design or syphilis stage) to explore heterogeneity were not feasible due to insufficient reporting of patient-level data in the primary studies. Similarly, meta-regression was not performed owing to the limited number of studies (< 10) for most outcomes. Sensitivity analyses were conducted by sequentially excluding individual studies.

Publication bias was assessed using funnel plots and Egger’s test (preferred for its higher statistical power than Begg’s test) when ≥10 studies were available, with the significance level set at α = 0.1 to enhance sensitivity given the typically low power of such tests. Forest plots were generated using the ‘meta’ package, and funnel plots were generated using ‘ggplot2’. A two-sided P < 0.05 was considered statistically significant for all analyses except publication bias testing.

The total number of studies included in the systematic review was 38. However, the sum of the number of studies across all meta-analyses exceeds this total. This is because many individual studies reported data on multiple immune indicators and were therefore included in more than one quantitative synthesis. For example, a study that measured both T-cell subsets and cytokine levels would be counted once in the overall study flow but contribute to both the ‘Lymphocyte’ and ‘Cytokine’ meta-analyses.

## 3 Results

### 3.1 Literature screening process

The initial database search yielded 1,352 records. Following deduplication and screening, 38 studies (35 in Chinese, 3 in English) involving 5,082 patients (1,900 in the serofast group) were included. The vast majority were case-control or cohort studies (37/38), predominantly conducted across multiple provinces of China. The overall risk of bias across the included studies was deemed acceptable​(7 low-risk, 28 moderate-risk, and 2 high-risk studies). The study selection process is detailed in the PRISMA flow diagram (**Figure 1**).

### 3.2 Cellular immune imbalance: CD4+ depletion, CD8+ dysregulation, and Th1/Th2 polarization

Meta-analysis of data from 10 studies revealed significant differences in peripheral blood lymphocyte subsets between patients with syphilis serofast (SF) and control groups (Healthy Controls, HC; Serologically Cured, SC) (**Table 2**).

**Table 2:** Meta-analysis of the association between peripheral blood lymphocytes and syphilis serofast.

Research factor	Number of studies	Study population	Statistical method	SMD (95%CI)	Heterogeneity test		
					Q	P	I²
CD4 (+)							
SF vs. HC	10	865	SMD (IV, FEM)	-0.61 (-0.75,-0.47)*	13.5	0.14	33.5%
SF vs. SC	7	652	SMD (IV, REM)	-0.73 (-1.13,-0.33)*	32.5	0.001	81.6%
CD8 (+)							
SF vs. HC	10	865	SMD (IV, REM)	0.40 (0.15,0.65)*	27.1	0.001	66.7%
SF vs. SC	7	652	SMD (IV, REM)	0.83 (0.19,1.46)*	72.9	0.001	91.8%
CD4 (+)/CD8 (+)							
SF vs. HC	5	391	SMD (IV, REM)	-0.44 (-0.80,-0.07)*	11.3	0.023	64.7%
SF vs. SC	3	314	SMD (IV, REM)	-0.42 (-1.54,0.71)	41.4	0.001	95.2%
NK cell							
SF vs. HC	7	622	SMD (IV, REM)	-0.85 (-1.35,-0.36)*	31.6	0.001	81.0%
SF vs. SC	4	404	SMD (IV, FEM)	-0.38 (-0.59,0.17)	3.4	0.331	12.2%
Th1 cell							
SF vs. HC Th2 cell	2	267	SMD (IV, FEM)	-0.61 (-0.86,-0.36)*	0.09	0.7653	0.0%
SF vs. HC	2	267	SMD (IV, FEM)	0.63 (0.27,0.99)*	1.66	0.1977	39.7%
Treg cell							
SF vs. HC	2	119	SMD (IV, FEM)	1.10 (0.71,1.48)*	0.1	0.758	0.0%

*P < 0.05; SF, Serofast Group; HC, Healthy Controls Group; SC, Serologically Cured Group.

Compared to the HC group, the SF group exhibited significantly lower levels of CD4+ T cells (SMD = -0.61, 95% CI: -0.75 to -0.47; I² = 33.5%) and a significantly higher levels of CD8+ T cells (SMD = 0.40, 95% CI: 0.15 to 0.65; I² = 66.7%). Consequently, the CD4+/CD8+ ratio was significantly inverted in the SF group (SMD = -0.44, 95% CI: -0.80 to -0.07; I² = 64.7%). In the comparison with the SC group, the SF group similarly showed significantly reduced CD4+ T cells (SMD = -0.73, 95% CI: -1.13 to -0.33; I² = 81.6%) and increased CD8+ T cells (SMD = 0.83, 95% CI: 0.19 to 1.46; I² = 91.8%). The difference in the CD4+/CD8+ ratio between SF and SC groups was not statistically significant (SMD = -0.42, 95% CI: -1.54 to 0.71; I² = 95.2%). Additionally, natural killer (NK) cell activity was significantly impaired in the SF group relative to the HC group (SMD = -0.85, 95% CI: -1.35 to -0.36; I² = 81.0%). No significant difference in NK cell activity was found between the SF and SC groups (SMD = -0.38, 95% CI: -0.59 to 0.17; I² = 12.2%). Complete data for all analyzed lymphocyte subsets are available in **Supplementary Table 4**.

### 3.3 Immune antibodies and complement

Meta-analysis of data from 8 studies revealed significant differences in peripheral blood inflammatory cytokine levels between patients with syphilis serofast (SF) and control groups (Healthy Controls, HC; Serologically Cured, SC) (**Table 3**).

**Table 3:** Meta-analysis of the association between immune antibodies, complement and syphilis serofast.

Research factor	Number of studies	Study population	Statistical method	SMD (95%CI)	Heterogeneity test		
					Q	P	I²
IgA							
SF vs. SC	3	577	SMD (IV, FEM)	-0.05 (-0.11,0.21)	3.80	0.1499	47.3%
IgM							
SF vs. SC	3	577	SMD (IV, REM)	0.96 (0.16,1.75)*	36.02	<0.001	94.4%
IgG							
SF vs. SC	2	459	SMD (IV, FEM)	0.35 (0.16,0.53)*	2.00	0.1578	49.9%
C3							
SF vs. SC	3	577	SMD (IV, REM)	-0.60 (-1.16,-0.04)*	18.11	0.0001	89.0%
C4							
SF vs. SC	3	577	SMD (IV, REM)	-0.80 (-1.35,0.25)	16.14	0.0003	87.6%

*P < 0.05; SF, Serofast Group; HC, Healthy Controls Group; SC, Serologically Cured Group.

Compared to the serologically cured (SC) group, the serofast (SF) group demonstrated significant humoral immune dysregulation characterized by distinct antibody and complement abnormalities. The SF group exhibited persistently elevated IgM levels (SMD = 0.96, 95% CI: 0.16 to 1.75) alongside significant complement consumption, as evidenced by reduced C3 (SMD = -0.60, 95% CI:-1.16 to -0.04 ) and C4 (SMD = -0.80, 95% CI: -1.35 to 0.35) levels. IgG levels were also modestly elevated (SMD = 0.35, 95% CI: 0.16 to 0.53).

### 3.4 Significant shift in Th1 and Th2 cytokines

The analysis of Th1 and Th2 cytokine levels demonstrated significant differences between groups (**Table 4**).

**Table 4:** Meta-analysis of the association between various cytokines and syphilis serofast.

Research factor	Number of studies	Study population	Statistical method	SMD (95%CI)	Heterogeneity test		
					Q	P	I²
Th2 cytokines							
IL-10							
SF vs. HC	9	695	SMD (IV, REM)	2.63 (1.61,3.64)*	106.3	0.001	92.5%
SF vs. SC	9	821	SMD (IV, REM)	2.17 (1.44,2.91)*	136.0	0.001	94.1%
SC vs. HC	8	713	SMD (IV, REM)	0.46 (0.09,0.83)*	39.4	0.001	82.2%
IL-4							
SF vs. HC	5	398	SMD (IV, REM)	2.92 (0.74,5.09)*	109.5	0.001	96.3%
SF vs. SC	6	474	SMD (IV, REM)	1.76 (0.69,2.84)*	75.7	0.001	93.4%
SCvs. HC	5	420	SMD (IV, REM)	0.79 (0.05,1.53)*	38.0	0.001	89.5%
Th1 cytokines							
IL-2							
SF vs. HC	6	456	SMD (IV, REM)	-2.25 (-3.47,-1.03)*	79.1	0.001	93.7%
SF vs. SC	7	534	SMD (IV, REM)	-2.11 (-3.16,-1.06)*	84.6	0.001	92.9%
IFN-γ							
SF vs. HC	5	471	SMD (IV, REM)	-2.19 (-3.70,-0.67)*	92.0	0.001	95.7%
SF vs. SC	6	682	SMD (IV, REM)	-1.56 (-2.95,-0.17)*	242.3	0.001	97.9%

*P < 0.05; SF, Serofast Group; HC, Healthy Controls Group; SC, Serologically Cured Group.

For Th1 cytokines, serum levels of IFN-γ were significantly lower in the SF group than in both the HC group (SMD = -2.19, 95% CI: -3.70 to -0.67; I² = 95.7%;) and the SC group (SMD = -1.56, 95% CI: -2.95 to -0.17; I² = 97.9%). Similarly, IL-2 levels were significantly reduced in the SF group compared to both HC (SMD = -2.25, 95% CI: -3.47 to -1.03; I² = 93.7%) and SC (SMD = -2.11, 95% CI: -3.16 to -1.06; I² = 92.9%) groups. Conversely, for Th2 cytokines, IL-10 levels were significantly elevated in the SF group compared to both HC (SMD = 2.63, 95% CI: 1.61 to 3.64; I² = 92.5%) and SC (SMD = 2.17, 95% CI: 1.44 to 2.91; I² = 94.1%) groups. IL-4 levels were also significantly higher in the SF group compared to HC (SMD = 2.92, 95% CI: 0.74 to 5.09; I² = 97.5%) and SC (SMD = 1.76, 95% CI: 0.69 to 2.84; I² = 95.3%) groups.

### 3.5 Toll-like receptors and chemokines

The meta-analysis of Toll-like receptors (TLRs) and chemokines included data from 6 studies. Compared to the healthy control (HC) group, the serofast (SF) group exhibited significantly lower expression of TLR2 mRNA (SMD = -1.52, 95% CI: -1.78 to -1.21; I² = 36.0%, p = 0.196). Compared to the serologically cured (SC) group, the SF group also showed significantly lower TLR2 mRNA expression (SMD = -1.03, 95% CI: -1.44 to -0.63; I² = 54.4%, p = 0.111) (**Table 5**). The serofast (SF) group exhibited significantly lower expression of TLR4 mRNA (SMD = -0.90, 95% CI: -1.22 to -0.59; I² = 0.0%, p = 0.381). The comparison between the SF group and the healthy control (HC) group for TLR4 mRNA showed no statistically significant difference (SMD = -1.02, 95% CI: -2.17 to 0.13; I² = 91.7%, p = 0.001). The CCR3 expression was significantly higher in the SF group compared to both the HC group (SMD = 4.29, 95% CI: 3.42 to 5.16) and the SC group (SMD = 4.69, 95% CI: 3.80 to 5.59), with no heterogeneity data as each comparison was based on a single study. For CXCR4, no significant differences were observed in any group comparisons (SF vs. HC: SMD = -0.05, 95% CI: -0.52 to 0.42; SF vs. SC: SMD = 0.28, 95% CI: -0.18 to 0.74; HC vs. SC: SMD = 0.35, 95% CI: -0.10 to 0.81), with all analyses based on single studies (**Supplementary Material S6**).

**Table 5:** Meta-analysis of the association between toll-like receptors, chemokines and syphilis serofast.

Research factor	Number of studies	Study population	Statistical method	SMD (95%CI)	Heterogeneity test		
					Q	P	I²
TLR2 mRNA							
SF vs. HC	4	399	SMD (IV,FEM)	-1.52 (-1.78,-1.21)*	4.7	0.196	36.0%
SF vs. SC	3	244	SMD (IV,REM)	-1.03 (-1.44,-0.63)*	4.4	0.111	54.4%
SC vs. HC	3	229	SMD (IV,REM)	-0.25 (-0.81,0.32)	9.5	0.009	79.0%
CCR5							
SF vs. HC	1	70	SMD (IV,FEM)	-2.76 (-3.42,-2.10)*	-	-	-
SF vs. SC	1	75	SMD (IV,FEM)	-2.74 (-3.38,-2.20)	-	-	-
HC vs. SC	1	75	SMD (IV,FEM)	-0.25 (-0.71,0.20)	-	-	-
CXCR3							
SF vs. HC	1	70	SMD (IV,FEM)	-3.66 (-4.44,-2.88)*	-	-	-
SF vs. SC	1	75	SMD (IV,FEM)	-5.00 (-5.94,-4.06)	-	-	-
HC vs. SC	1	75	SMD (IV,FEM)	-0.84 (-1.32,-0.37)	-	-	-

*P < 0.05; SF, Serofast Group; HC, Healthy Controls Group; SC, Serologically Cured Group.

### 3.6 miRNA

Due to the limited number of available studies for each specific miRNA, a quantitative meta-analysis was not feasible. However, the findings from individual studies are summarized descriptively below and detailed in **Supplementary Material S7**.

Elevated Expression: Compared to healthy controls (HC), patients with syphilis serofast (SF) showed higher expression levels of miR-146a, miR-155, miR-299-3P, miR-195, miR-223, and miR-589 in the studies where they were measured. Reduced Expression: The SF group exhibited lower expression levels of miR-31 and miR-192 compared to the HC group.

Each of the above comparisons is based on a single study. The standardized mean differences (SMD) with 95% confidence intervals for all reported miRNAs, including comparisons with serologically cured (SC) groups, are provided in **Supplementary Material S7**.

### 3.7 Heterogeneity explanation

High heterogeneity was observed in analyses of CD4+/CD8+ ratios, Treg cells, IgM, C3, C4, Th1, and Th2 cytokines. This heterogeneity stemmed from differences in patient disease duration and treatment backgrounds across studies, or from variations in detection methods (e.g., reagents from different manufacturers). Therefore, a random-effects model was employed to pool all cytokine analyses, yielding estimates that reflect the distribution of “average effects” rather than a single “true effect.” The overall trend remains statistically and biologically significant.

### 3.8 Sensitivity analysis and publication bias assessment

Sensitivity analyses confirmed the robustness of the primary findings for CD4+/CD8+ ratio imbalance and Th1/Th2 cytokine bias. Given the scarcity of literature evaluating specific factors in relation to study outcomes, publication bias was assessed only for factors with ≥6 included studies. Results showed generally symmetrical funnel plots, with no apparent publication bias observed at first glance. As funnel plots represent a qualitative assessment method, different observers may reach varying conclusions.

Therefore, we also employed Begg’s test, Egger’s test, and Macaskill’s test to assess publication bias. Results showed no significant asymmetry in any group at the α=0.1 threshold, indicating no evidence of significant publication bias.

## 4 Discussion

**Figure 2:**
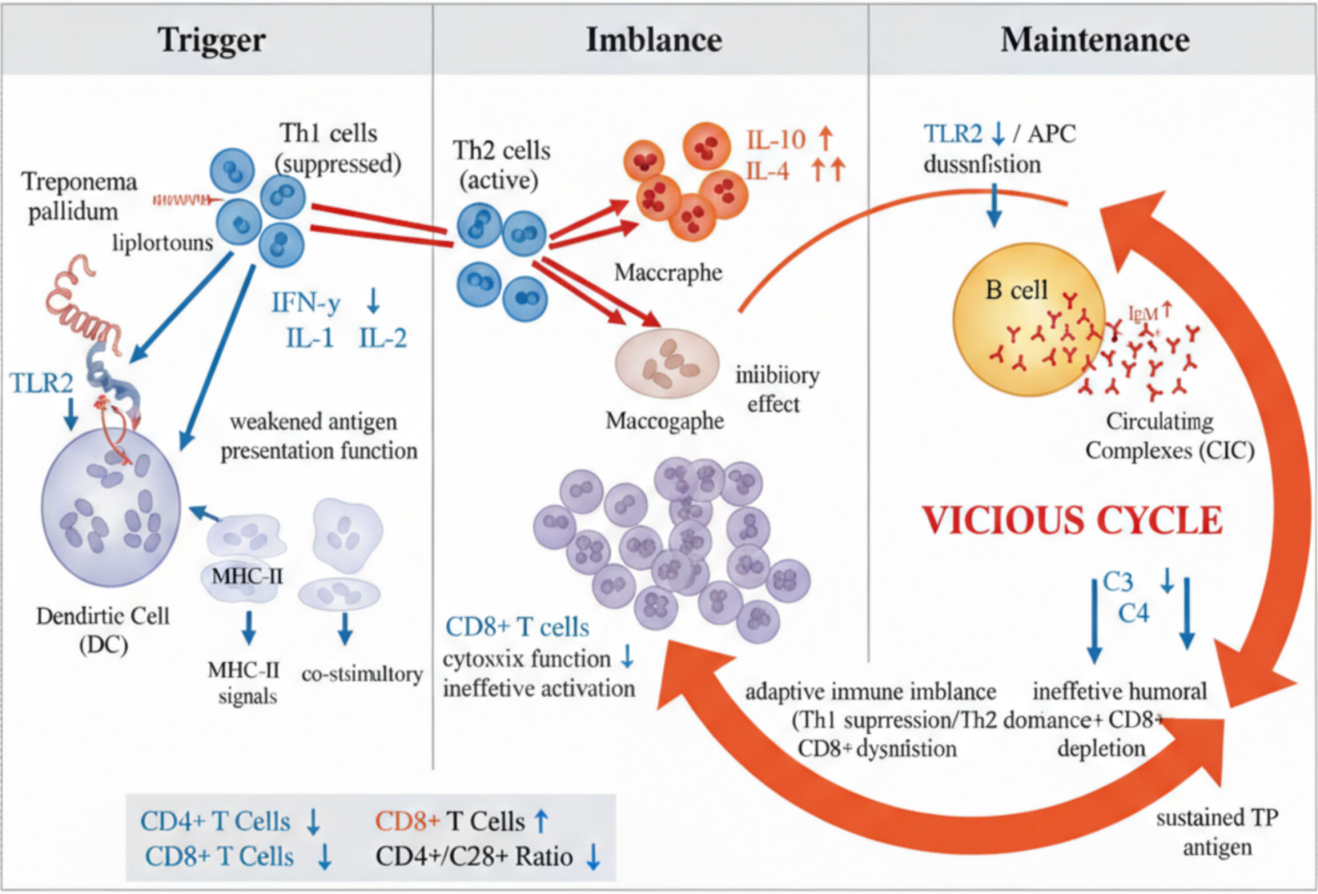
Immunological mechanism of the serofast in syphilis (image created from BioGDP) This model illustrates the self-perpetuating immune dysfunction underlying the serofast. The process is initiated by impaired innate immune recognition of Treponema pallidum (TP) due to Toll-like receptor 2 (TLR2) downregulation, leading to an adaptive immune imbalance characterized by a suppressed Th1 response (e.g., decreased IFN-γ, IL-2) and a dominant Th2 response (e.g., elevated IL-10). The high IL-10 levels inhibit effective macrophage and CD8+ T-cell function, preventing the clearance of persistent TP antigens and resulting in a vicious cycle that maintains low-level seropositivity. Key meta-analysis findings supporting this model are integrated. DC, dendritic cell; TLR2, Toll-like receptor 2; Th, T helper cell; IFN-γ, interferon-gamma; IL, interleukin.

Our meta-analysis culminates in an integrated immunopathological model that delineates the self-perpetuating cascade underlying the serofast, as depicted in **Figure 2**. This model proposes a sequential cascade initiated by impaired innate immune recognition, leading to ​polarized adaptive immunity, and culminating in a vicious cycle of humoral dysregulation and complement exhaustion, which collectively prevent the clearance of Treponema pallidum(TP) and sustain low-grade seropositivity. The following sections will dissect each component of this model, underpinning them with our quantitative findings.

### 4.1 Failure of innate immune recognition: the primary trigger mechanism in serofast

Quantitative evidence from this meta-analysis indicates a significant downregulation of Toll-like receptor 2 (TLR2) expression in serum-fixed patients (SMD = -1.46). This not only distinguishes them from the serum-cured group but also represents a primary failure of innate immune recognition. Studies by Brightbill, H. D., Shin, J. S., et al. (24, 27, 28) explicitly describe how Treponema pallidum lipoproteins activate inflammation via the TLR2 pathway. TLR2 serves as a crucial “gatekeeper” receptor recognizing pathogen-associated molecular patterns (PAMPs) such as lipoproteins on Treponema pallidum (TP) (29). Its downregulation directly impairs antigen-presenting cells (e.g., dendritic cells, DCs) in recognizing TP, preventing the effective transmission of the “first signal” required for activating the adaptive immune response and the co-stimulatory “second signal.” This fundamentally weakens the initiation intensity of subsequent T-cell and B-cell responses, creating preconditions for early immune escape and persistent latency of TP. This discovery traces the root cause of serum-fixed immunodeficiency to the innate immune level, offering a new perspective on understanding its persistence.

Therefore, the significant downregulation of TLR2 (SMD= -1.46) is not merely an associated finding but may represent the initial defect​in our proposed model (**Figure 2**), compromising the very foundation of immune recognition and setting the stage for the subsequent adaptive immune failures.

### 4.2 Polarization of adaptive immunity

Building upon impaired innate immune recognition, this study quantifies profound polarization of adaptive immunity. First, serum-fixed patients exhibit a pattern of CD4+ T cell exhaustion, dysfunctional activation of CD8+ T cells, and an inverted CD4+/CD8+ ratio (see Section 2.1). The significant reduction in their numbers strongly suggests the presence of “T cell exhaustion” (30) Persistent antigen stimulation led to increased CD8+ T cell numbers, but this expansion was accompanied by dysfunction, losing effective cytotoxicity and the ability to produce effector molecules (such as IFN-γ and perforin), representing a state of “ineffective activation.” The inverted CD4+/CD8+ ratio signifies the immune system shifting from a normal synergistic state into a contradictory state of “coexisting suppression and chronic activation.”

Furthermore, serum-fixed patients exhibit a marked Th1/Th2 imbalance consistent with Arlene C Seña’s findings (31), characterized by severe suppression of Th1 cytokines (e.g., IFN-γ, SMD = -2.33;IL-2, SMD = -1.83) and abnormal predominance of Th2 cytokines (e.g., IL-10, SMD = +1.97; IL-4, SMD = +2.92). This represents not merely a shift in immune response type but an active immunosuppressive process:

• Th1 dysfunction: Reduced IL-2 impairs the effective expansion of antigen-specific T cell clones.IL-2 plays multiple critical roles in immune responses: amplifying antigen-specific clones (32), sustaining effector T cell function (33, 34), and regulating immune memory formation (35, 36). Consequently, the deficiency of IL-2 *in vivo* (SMD = -2.11) may represent a core defect leading to “failed initiation” of cellular immunity and the inability to establish long-lasting immune memory. Reduced IL-2 further diminishes IFN-γ secretion by Th1 cells. Extreme IFN-γ deficiency prevents effective macrophage activation, eliminating the critical effector mechanism for clearing intracellular TP.

• Th2 Suppression: Crucially, elevated levels of IL-10—a core immunosuppressive factor—actively maintain an inhibitory immune microenvironment by directly suppressing macrophage activation and Th1 cell function. In the clinical study by Pastuszczak et al. (37), serum-stabilized patients exhibited higher IL-10 levels compared to serum-cured patients. Active Th2 suppression was also noted in studies by Fitzgerald, T J (15, 38). Concurrently, dysregulation of chemokine receptor networks—such as upregulation of CCR3 and downregulation of CXCR3—systemically recruits Th2 cells to infection sites while impeding the recruitment of Th1 cells and cytotoxic T cells, spatially cementing this Th2-biased microenvironment. This polarization and imbalance collectively lead to failure of the cellular immune response. Although Fitzgerald et al. (15) previously proposed the concept of Th1/Th2 drift, our study provides the first quantitative confirmation through meta-analysis of large-scale data, revealing the central role of IL-10 (SMD = +1.97) as a key immunosuppressive factor, thereby advancing this hypothesis to the empirical stage.

### 4.3 Humoral immune dysregulation, complement depletion, and vicious cycle

Immune polarization further triggers cascading dysregulation of humoral immunity and the complement system, ultimately forming a vicious cycle that perpetuates serofast:

- Abnormal antibody response: Manifested as persistently elevated IgM levels (SMD = +0.96), strongly suggesting persistent presence and repeated stimulation by TP antigens, disrupting the conventional IgG-dominant pattern in chronic infections.
- Complement System Depletion: Marked reductions in C3 (SMD = -0.60) and C4 (SMD = -0.80) demonstrate excessive and sustained activation and depletion of the complement system by immune complexes (ICs). These ICs, formed by TP antigens and corresponding antibodies (primarily IgM), activate the classical complement pathway, leading to cleavage and depletion of complement components. Depletion of the complement system makes complete clearance of pathogens more difficult (39, 40), further contributing to serofast.
- Formation of a vicious cycle: The aforementioned processes constitute a self-perpetuating pathological cycle: “Persistent TP → Stimulation of IgM production → Formation of circulating immune complexes (CIC) → Activation and depletion of complement → Complement depletion weakens CIC and pathogen clearance capacity → More persistent TP.” This cycle traps the immune system in a “stalemate” state where it cannot completely clear the pathogen nor fully resolve inflammation, explaining the persistence and treatment resistance of serofast.

This series of events—persistent antigen → IgM → CIC →complement consumption → failed clearance—forms the self-sustaining, vicious cycle that is the hallmark of our model (**Figure 2**). It explains not just the immune dysfunction but the chronicity of the serofast, trapping the host in an immunological stalemate.

### 4.4 Broader implications and international context

The immunopathological cascade elucidated in our model—triggered by impaired TLR2-mediated recognition and amplified by a dominant IL-10 response—exhibits striking similarities to other well-studied models of persistent infection. In tuberculosis (a classic example of intracellular persistent infection), IL-10-mediated immunosuppression (41–44) has been identified as a mechanism promoting pathogen survival. Similarly, in HIV infection, the pathogen downregulates TLR2 and induces an IL-10-dominant anti-inflammatory response (45); In hepatitis C, HCV achieves immune evasion and persistence by triggering TLR2-mediated monocyte activation via HCV core protein, inducing cytokines that cause PDC apoptosis and suppress IFN-α production (28) thereby inhibiting host immune expression and promoting disease chronicity.

### 4.5 Implications of the study’s broad significance and geographic distribution characteristics

Among the 38 studies included in this research, 35 originated from Chinese populations, objectively reflecting the current state of the global evidence base in this specific field. This concentration is not due to search bias but rather because international studies predominantly focus on epidemiology, clinical outcomes, or co-infections, with most endpoints being the dichotomous outcome of “serological cure.” In contrast, domestic research delves deeper into the immunopathological mechanisms underlying serofast, systematically reporting quantitative immunological data suitable for meta-analysis. Consequently, this meta-analysis represents the first systematic synthesis of the best available evidence.

Despite the relatively concentrated evidence sources, the immune cascade reaction revealed in this study—centered on “cellular immunosuppression-Th1/Th2 shift-complement depletion”—holds significant universal implications. This immune signature bears striking similarities to the IL-10-dominant immunosuppression patterns observed in other chronic infections such as tuberculosis and HIV infection (34–39), suggesting it may represent a common pathway of immune persistence across pathogens and populations. Our findings provide a critical theoretical framework and quantitative baseline for understanding the core mechanisms of serofast.

Building upon this foundation, this study holds clear translational medicine and public health value. The identified key immune markers (e.g., elevated IL-10, low CD4+/CD8+ ratio) hold potential as stratification biomarkers to distinguish serofast patients who may benefit from escalating from observation-based management to immunomodulatory interventions. This approach advances precision medicine for syphilis and optimizes healthcare resource allocation.

## 5 Limitations and future directions

Of course, we must acknowledge the limitations of this study. The primary limitation lies in the geographic specificity of the evidence, as the findings are primarily based on Chinese populations. This reflects the current state of knowledge and points to a key direction for future research: the need to validate the universality of this immune signature across different ethnicities, regions, and TP subtypes.

Additionally, this study has several limitations: First, most included studies failed to distinguish between early and late latent syphilis, potentially obscuring the heterogeneous impact of disease stage on immune signatures. Second, dynamic post-treatment follow-up data were lacking for most immune markers, hindering the characterization of their trajectory. Finally, inadequate reporting of potential sources of heterogeneity (e.g., assay kits, precise disease stages) in original studies prevented quantitative exploration via meta-regression or similar methods. Furthermore, the high heterogeneity observed in several meta-analyses could not be fully explored through pre-specified subgroup analyses (e.g., by syphilis stage or patient sex). This was primarily due to the inconsistent and insufficient reporting of these key variables in the original studies. This limitation highlights an important issue in the current literature on syphilis serofast and underscores the need for more standardized and comprehensive reporting of patient demographics and clinical characteristics in future research.

We call on global colleagues to standardize reporting of key quantitative immunological data in syphilis outcome studies to build more universally applicable models. Future research should focus on: 1) Establishing multinational, multicenter prospective cohorts to validate the universality of identified immune biomarkers; 2) Deepening investigations into the roles of TP genetic diversity, dendritic cell antigen presentation function, and immune cell apoptosis pathways in serofast; 3) Ultimately translating these mechanistic discoveries into point-of-care diagnostic tools and integrating immune status assessment into syphilis management guidelines, thereby offering novel strategies to address this clinical challenge.

## 6 Conclusion

This meta-analysis establishes that the syphilis serofast is driven by a coherent immunopathological cascade. The process is initiated by a failure of innate immune recognition, propagates into a profoundly polarized adaptive response characterized by Th1 suppression and dominant Th2 activity, and culminates in a self-sustaining cycle of humoral dysregulation and complement exhaustion.

Our findings provide a definitive immunological basis for serofast persistence, highlighting the central roles of IL-10 overproduction and Th1 cytokine deficiency. This evidence positions the reversal of this specific immune imbalance as a promising therapeutic strategy, advocating for future exploration of immunomodulatory interventions adjunctive to standard antibiotic therapy.

## Data Availability

The original contributions presented in the study are included in the article/**Supplementary Material**. Further inquiries can be directed to the corresponding authors.
