# Supplementary material for: An integrated immunopathological model of syphilis serofast: a systematic review and meta-analysis
Source: Front Immunol. 2026 Mar 30;17:1758075. doi: 10.3389/fimmu.2026.1758075 (PMC13070755; doi:10.3389/fimmu.2026.1758075)
Supplement: Supplementary file 1 [file DataSheet1.docx]

****Supplemental Material S1****

****Supplemental Material S1.** Search Strategies**

| **Databases** | **Term** |
| --- | --- |
| PubMed、Google Scholar、Web of Science | "Syphilis"[Mesh] OR "Treponema pallidum"[Mesh] OR syphilis[tiab] OR treponem*[tiab] OR lues[tiab] |
|  | serofast[tiab] OR "sero-resistance"[tiab] OR "serological nonresponse"[tiab] OR "serofast"[tiab] OR ((serolog*[tiab] OR RPR[tiab] OR VDRL[tiab]) AND (fix*[tiab] OR resistan*[tiab] OR nonrespon*[tiab])) |
|  | "Immunity"[Mesh] OR "Immune System"[Mesh] OR immune[tiab] OR immun*[tiab] OR "T lymphocyte"[tiab] OR "T cell"[tiab] OR cytokine[tiab] OR "CD4"[tiab] OR "CD8"[tiab] OR "NK cell"[tiab] OR "Dendritic Cells"[Mesh] OR "Toll-Like Receptors"[Mesh] OR "MicroRNAs"[Mesh] |
|  | #1 AND #2 AND #3 |
| CNKI、WanFang Data、VIP、CBMdisc | **（梅毒 OR 螺旋体）AND （血清固定 OR 血清抵抗） AND (免疫 OR 淋巴细胞 OR 细胞因子** |
|  | **血清固定 OR 血清抵抗 OR 血清不反应 OR （血清学试验 AND 固定） OR (RPR AND 固定） OR (TRUST AND 固定）** |
|  | **主题词：免疫/全部树/全部副主题词 OR 主题词：T淋巴细胞/全部树/全部副主题词 OR 主题词：细胞因子/全部树/全部副主题词 OR 免疫 OR 淋巴细胞 OR 细胞因子 OR CD4 OR CD8 OR NK细胞 OR 树突细胞 OR Toll样受体 OR 微小RNA** |

**Search Date: 2024/12/31**

****Supplemental Material S2****


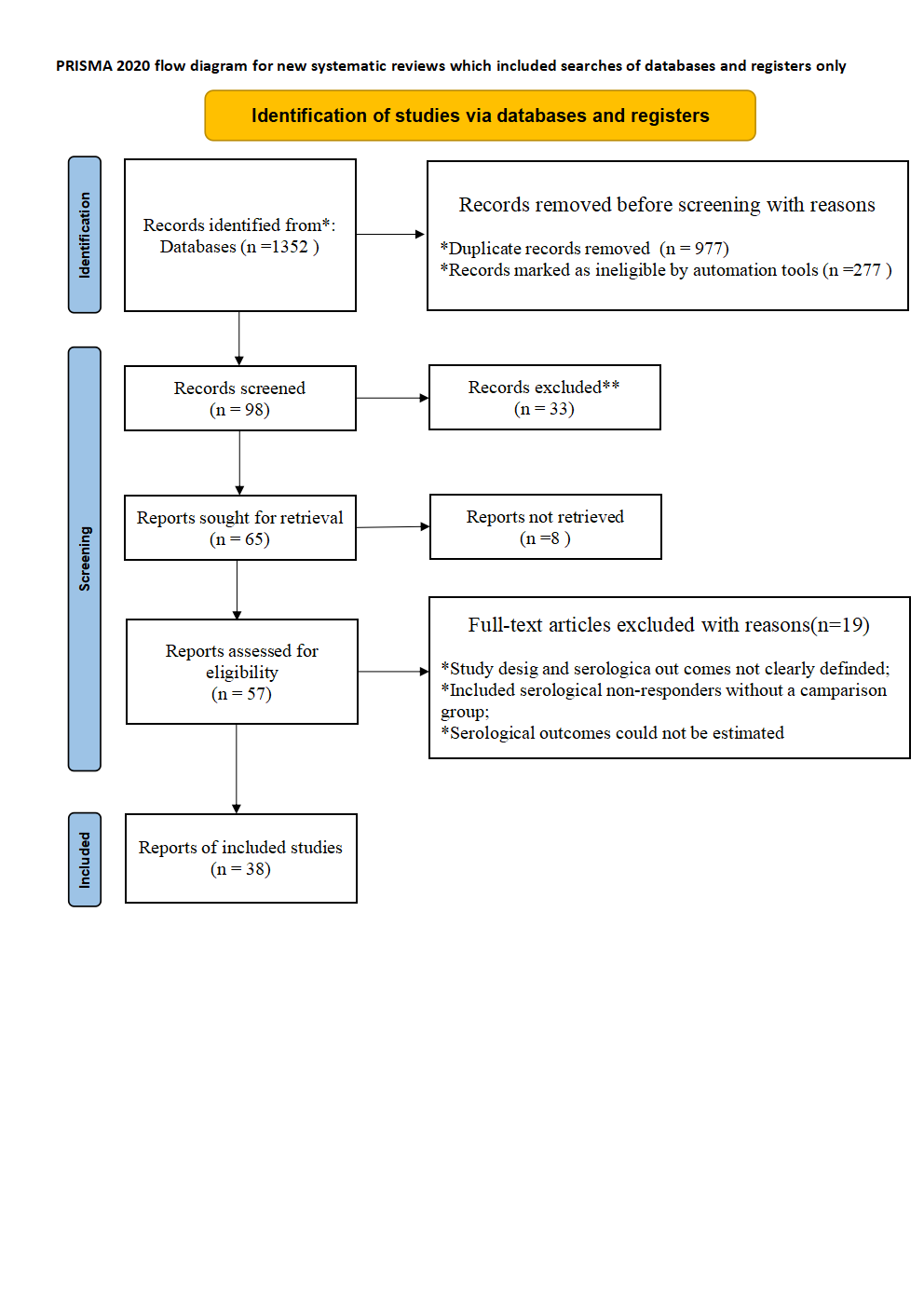


**Fig. 1** PRISMA flow diagram of the systematic review process using the terms “syphilis” and “serology” and “resistance”, “response”, “serofast”,or “seroresistance” in seven databases.

****Supplemental Material S3****


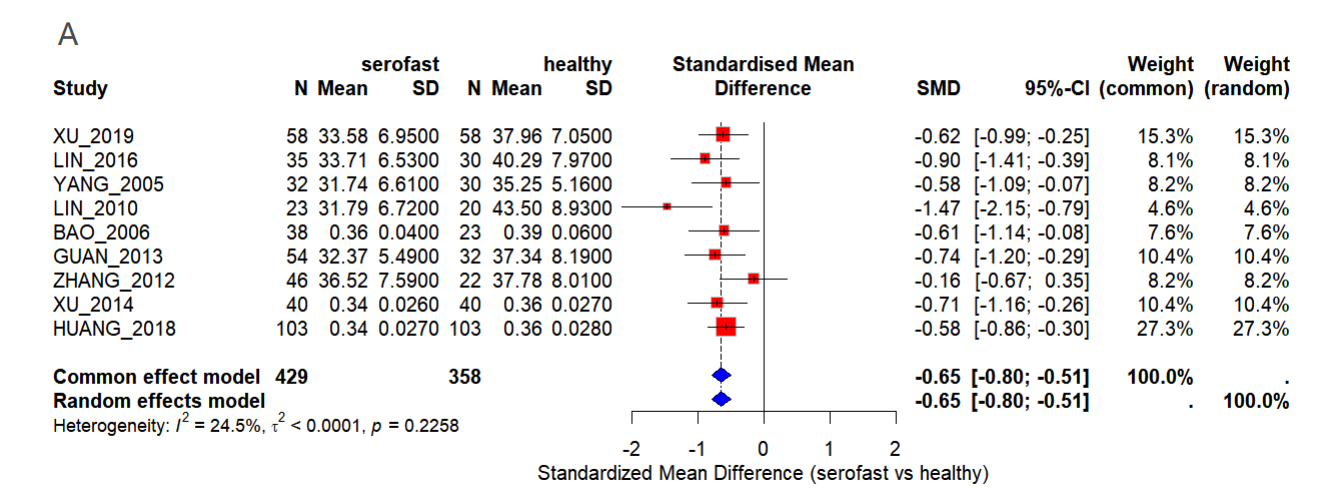


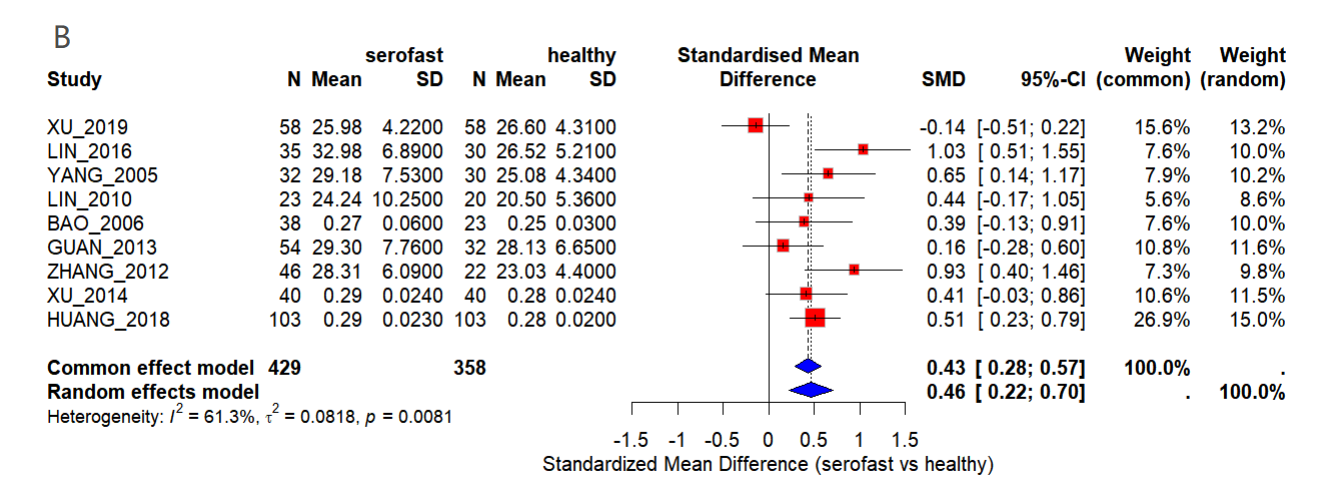


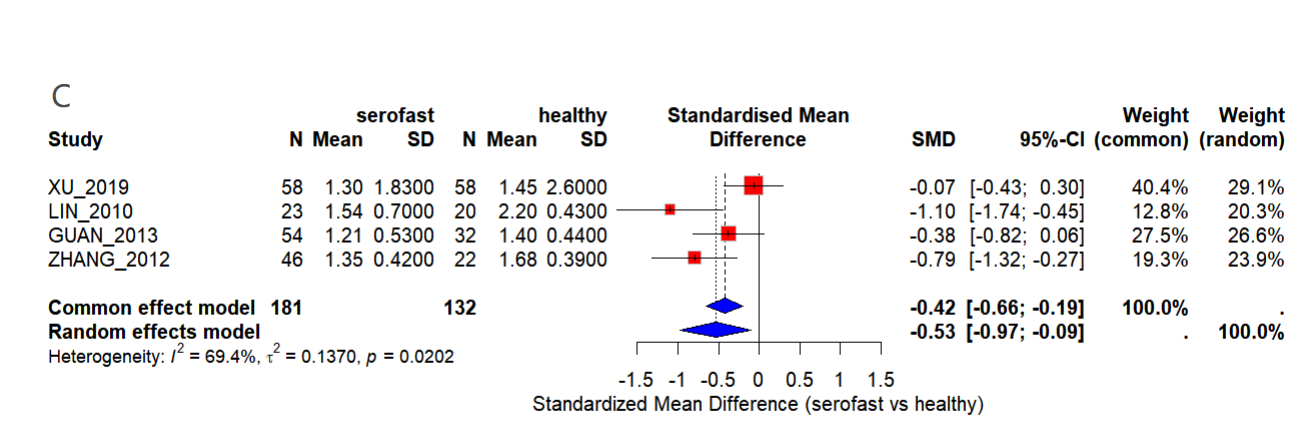


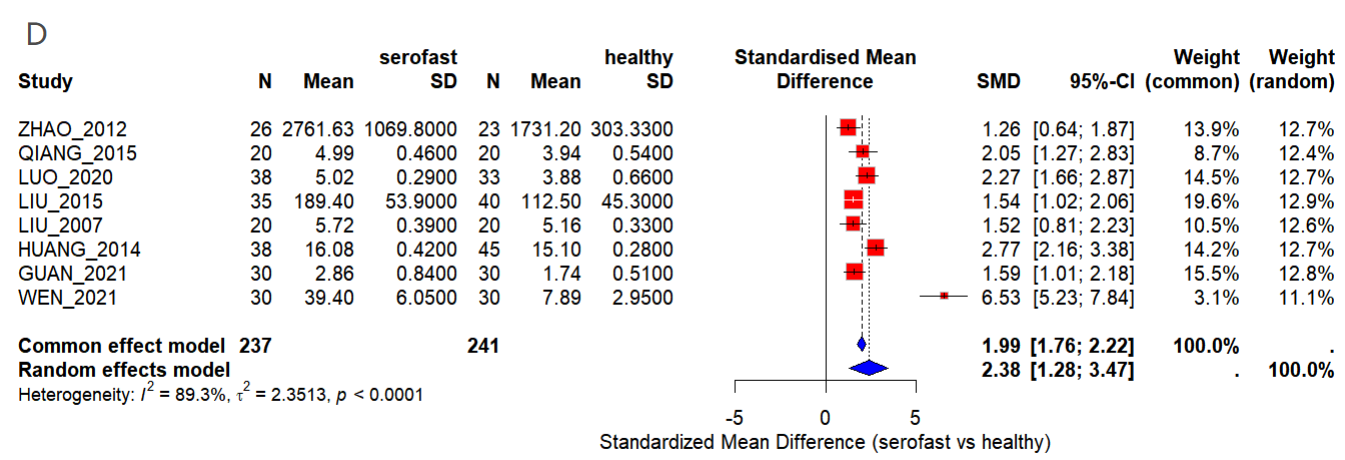


**Fig 2.**Sensitivity analysis of serofast Immunological factors (A:CD4, B:CD8, C:CD4/CD8, D:IL-10)

****Supplemental Material S4****.Meta-analysis of the Association Between Peripheral Blood Lymphocytes and Syphilis Serofast

| Research Factor | Number of Studies | Study Population | Statistical Method | SMD(95%CI) | Heterogeneity Test | | |
| --- | --- | --- | --- | --- | --- | --- | --- |
|  |  |  |  |  | Q | P | I² |
| CD3（+） |  |  |  |  |  |  |  |
| SF vs. HC | 9 | 782 | SMD(Ⅳ, FEM) | -0.01(-0.15,0.13) | 14.9 | 0.061 | 46.3% |
| SF vs. SC | 3 | 251 | SMD(Ⅳ, FEM) | -0.13(-0.40,0.10) | 2.6 | 0.274 | 22.7% |
| SC vs. HC | 2 | 130 | SMD(Ⅳ, REM) | -0.27(-0.85,0.31) | 2.4 | 0.121 | 58.3% |
| CD4（+） |  |  |  |  |  |  |  |
| SF vs. HC | 10 | 865 | SMD(Ⅳ, FEM) | -0.61(-0.75,-0.47)* | 13.5 | 0.14 | 33.5% |
| SF vs. SC | 7 | 652 | SMD(Ⅳ, REM) | -0.73(-1.13,-0.33)* | 32.5 | 0.001 | 81.6% |
| SC vs. HC | 4 | 254 | SMD(Ⅳ, REM) | -0.40(-1.26,0.47) | 21.4 | 0.001 | 86.0% |
| CD8（+） |  |  |  |  |  |  |  |
| SF vs. HC | 10 | 865 | SMD(Ⅳ, REM) | 0.40(0.15,0.65)* | 27.1 | 0.001 | 66.7% |
| SF vs. SC | 7 | 652 | SMD(Ⅳ, REM) | 0.83(0.19,1.46)* | 72.9 | 0.001 | 91.8% |
| SC vs. HC | 4 | 254 | SMD(Ⅳ, FEM) | 0.19(-0.05,0.44) | 2.2 | 0.541 | 0.0% |
| CD4（+）/CD8（+） |  |  |  |  |  |  |  |
| SF vs. HC | 5 | 391 | SMD(Ⅳ, REM) | -0.44(-0.80,-0.07)* | 11.3 | 0.023 | 64.7% |
| SF vs. SC | 3 | 314 | SMD(Ⅳ, REM) | -0.42(-1.54,0.71) | 41.4 | 0.001 | 95.2% |
| SC vs. HC | 2 | 99 | SMD(Ⅳ, REM) | -0.98(-2.72,-0.75) | 14.4 | 0.001 | 93.1% |
| NK cell |  |  |  |  |  |  |  |
| SF vs. HC | 7 | 622 | SMD(Ⅳ, REM) | -0.85(-1.35,-0.36)* | 31.6 | 0.001 | 81.0% |
| SF vs. SC | 4 | 404 | SMD(Ⅳ, FEM) | -0.38(-0.59,0.17) | 3.4 | 0.331 | 12.2% |
| SC vs. HC | 3 | 189 | SMD(Ⅳ, REM) | -0.38(-0.99,0.23) | 7.0 | 0.031 | 71.3% |
| B cell |  |  |  |  |  |  |  |
| SF vs. HC | 5 | 387 | SMD(Ⅳ, FEM) | 0.04(-0.16,0.24) | 4.13 | 0.3884 | 3.2% |
| SF vs. SC | 2 | 120 | SMD(Ⅳ, REM) | 0.07(-0.51,0.65) | 2.36 | 0.1247 | 57.6% |
| Th1 cell |  |  |  |  |  |  |  |
| SF vs. HC | 2 | 267 | SMD(Ⅳ, FEM) | -0.61(-0.86,-0.36)* | 0.09 | 0.7653 | 0.0% |
| Th2 cell |  |  |  |  |  |  |  |
| SF vs. HC | 2 | 267 | SMD(Ⅳ, FEM) | 0.63(0.27,0.99)* | 1.66 | 0.1977 | 39.7% |
| Tc cell |  |  |  |  |  |  |  |
| SF vs. HC | 2 | 267 | SMD(Ⅳ, FEM) | -0.62(-0.87,-0.37) | 0.42 | 0.5173 | 0.0% |
| Ts cell |  |  |  |  |  |  |  |
| SF vs. HC | 2 | 267 | SMD(Ⅳ, REM) | 0.80(0.33,1.27)* | 2.43 | 0.1187 | 58.9% |
| Treg cell |  |  |  |  |  |  |  |
| SF vs. HC | 2 | 119 | SMD(Ⅳ, FEM) | 1.10(0.71,1.48)* | 0.1 | 0.758 | 0.0% |

SF:Serofast Group;HC:Healthy Controls Group;SC:Serologically Cured Group

**P*<0.05

****Supplemental Material S5.**Comprehensive Meta-Analysis Results of Serum Cytokine Levels in Syphilis Serofast Patients.**

| Research Factor | ​Number of Studies | Study Population | Statistical Method | SMD(95%CI) | Heterogeneity Test | | | | |
| --- | --- | --- | --- | --- | --- | --- | --- | --- | --- |
|  |  |  |  |  | Q | P | | I² | |
| **Th2 cytokines** |  |  |  |  |  | |  | |  |
| IL-10 |  |  |  |  |  | |  | |  |
| SF vs. HC | 9 | 695 | SMD(Ⅳ, REM) | 2.63(1.61,3.64)* | 106.3 | | 0.001 | | 92.5% |
| SF vs. CS | 9 | 821 | SMD(Ⅳ, REM) | 2.17(1.44,2.91)* | 136.0 | | 0.001 | | 94.1% |
| CS vs. HC | 8 | 713 | SMD(Ⅳ, REM) | 0.46(0.09,0.83)* | 39.4 | | 0.001 | | 82.2% |
| CS vs. SP | 5 | 346 | SMD(Ⅳ, REM) | -0.34(-1.33,0.64) | 77.1 | | 0.001 | | 94.8% |
| IL-4 |  |  |  |  |  | |  | |  |
| SF vs. HC | 5 | 398 | SMD(Ⅳ, REM) | 2.92(0.74,5.09)* | 109.5 | | 0.001 | | 96.3% |
| SF vs. CS | 6 | 474 | SMD(Ⅳ, REM) | 1.76(0.69,2.84)* | 75.7 | | 0.001 | | 93.4% |
| CS vs. HC | 5 | 420 | SMD(Ⅳ, REM) | 0.79(0.05,1.53)* | 38.0 | | 0.001 | | 89.5% |
| CS vs. SP | 5 | 366 | SMD(Ⅳ, REM) | -0.64(-1.31,0.03) | 37.3 | | 0.001 | | 89.3% |
| IL-18 |  |  |  |  |  | |  | |  |
| SF vs. HC | 2 | 218 | SMD(Ⅳ, REM) | -0.21(-5.54,5.13) | 202.2 | | 0.001 | | 99.5% |
| IL-6 |  |  |  |  |  | |  | |  |
| SF vs. HC | 4 | 363 | SMD(Ⅳ, REM) | -0.66(-1.97,0.64) | 68.4 | | 0.001 | | 95.6% |
| SF vs. CS | 4 | 477 | SMD(Ⅳ, REM) | -0.68(-1.00,-0.36)* | 7.7 | | 0.053 | | 60.9% |
| CS vs. HC | 4 | 430 | SMD(Ⅳ, REM) | 0.06(-1.11,1.23) | 56.1 | | 0.001 | | 94.7% |
| CS vs. SP | 3 | 197 | SMD(Ⅳ, REM) | -0.86(-2.41,0.69) | 46.1 | | 0.001 | | 95.7% |
|  |  |  |  |  |  | |  | |  |
| **Th1 cytokines** |  |  |  |  |  | |  | |  |
| IL-2 |  |  |  |  |  | |  | |  |
| SF vs. HC | 6 | 456 | SMD(Ⅳ, REM) | -2.25(-3.47,-1.03)* | 79.1 | | 0.001 | | 93.7% |
| SF vs. CS | 7 | 534 | SMD(Ⅳ, REM) | -2.11(-3.16,-1.06)* | 84.6 | | 0.001 | | 92.9% |
| CS vs. HC | 6 | 480 | SMD(Ⅳ, REM) | -0.05(-0.41,0.31) | 16.9 | | 0.005 | | 70.3% |
| CS vs. SP | 5 | 366 | SMD(Ⅳ, REM) | 0.78(0.10,1.46) | 32.0 | | 0.001 | | 87.5% |
| IFN-γ |  |  |  |  |  | |  | |  |
| SF vs. HC | 5 | 471 | SMD(Ⅳ, REM) | -2.19(-3.70,-0.67)* | 92.0 | | 0.001 | | 95.7% |
| SF vs. CS | 6 | 682 | SMD(Ⅳ, REM) | -1.56(-2.95,-0.17)* | 242.3 | | 0.001 | | 97.9% |
| CS vs. HC | 5 | 533 | SMD(Ⅳ, REM) | -0.10(-0.67,0.47) | 42.2 | | 0.001 | | 90.5% |
| CS vs. SP | 3 | 259 | SMD(Ⅳ, REM) | 0.68(-0.29,1.64) | 28.1 | | 0.001 | | 92.9% |
| IL-12 |  |  |  |  |  | |  | |  |
| SF vs. HC | 6 | 398 | SMD(Ⅳ, REM) | -1.40(-2.81,0.02) | 100 | | 0.001 | | 95.0% |
| SF vs. CS | 6 | 341 | SMD(Ⅳ, REM) | -1.58(-3.04,-0.12)* | 80.2 | | 0.001 | | 93.8% |
| CS vs. HC | 5 | 299 | SMD(Ⅳ, FEM) | -0.12(-0.47,0.17) | 5.9 | | 0.208 | | 32.1% |
| CS vs. SP | 5 | 253 | SMD(Ⅳ, REM) | 0.48(0.23,0.73) | 5.4 | | 0.249 | | 25.9% |
| TNF-α |  |  |  |  |  | |  | |  |
| SF vs. HC | 2 | 148 | SMD(Ⅳ, REM) | -2.55(-6.24,1.13) | 59 | | 0.0001 | | 98.3% |
| SF vs. CS | 2 | 142 | SMD(Ⅳ, REM) | -2.09(-4.98,0.80) | 41.4 | | 0.0001 | | 97.6% |
| CS vs. HC | 2 | 144 | SMD(Ⅳ, REM) | -0.36(-0.89,0.18) | 2.6 | | 0.109 | | 61.0% |
| CS vs. SP | 2 | 131 | SMD(Ⅳ, FEM) | -0.63(-0.98,-0.28)* | 0.0 | | 0.836 | | 0.0% |
|  |  |  |  |  |  | |  | |  |
| **Th17 cytokines** |  |  |  |  |  | |  | |  |
| IL-17 |  |  |  |  |  | |  | |  |
| SF vs. CS | 3 | 210 | SMD(Ⅳ, FEM) | -1.28(-1.58,-0.98)* | 0.4 | | 0.813 | | 0.0% |
| CS vs. HC | 2 | 120 | SMD(Ⅳ, FEM) | 0.10(-0.26,0.46) | 0.1 | | 0.7699 | | 0.0% |
| CS vs. SP | 2 | 120 | SMD(Ⅳ, REM) | -1.78(-5.50,1.93) | 56.4 | | 0.001 | | 98.2% |
| IL-22 |  |  |  |  |  | |  | |  |
| SF vs. CS | 2 | 120 | SMD(Ⅳ, REM) | -1.16(-2.04,-0.29)* | 4.9 | | 0.0264 | | 79.7% |
|  |  |  |  |  |  | |  | |  |
| **Non-specific inflammatory mediators** |  |  |  |  |  | |  | |  |
| IL-1β |  |  |  |  |  | |  | |  |
| SF vs. HC | 2 | 218 | SMD(Ⅳ, REM) | -1.63(-2.83,6.10) | 144.7 | | 0.001 | | 99.3% |
|  |  |  |  |  |  | |  | |  |
| **Regulatory cytokines** |  |  |  |  |  | |  | |  |
| TGF-β |  |  |  |  |  | |  | |  |
| SF vs. HC | 3 | 188 | SMD(Ⅳ, REM) | 1.35(-0.50,3.20) | 49 | | 0.001 | | 95.9% |
| SF vs. CS | 3 | 200 | SMD(Ⅳ, REM) | 0.81(-0.28,1.89) | 27 | | 0.001 | | 92.6% |
| CS vs. HC | 3 | 192 | SMD(Ⅳ, REM) | 0.65(-0.26,1.55) | 17.8 | | 0.001 | | 88.8% |
| CS vs. SP | 2 | 132 | SMD(Ⅳ, REM) | 0.37(-0.49,1.22) | 5.9 | | 0.015 | | 83.1% |

SF:Serofast Group;HC:Healthy Controls Group;SC:Serologically Cured Group;SP:Syphilis Patients

**P*<0.05

****Supplemental Material S6.**** Meta-analysis of the Association Between Toll-like Receptors, Chemokines and Syphilis Serofast

| Research Factor | ​Number of Studies | Study Population | Statistical Method | SMD(95%CI) | Heterogeneity Test | | | | |
| --- | --- | --- | --- | --- | --- | --- | --- | --- | --- |
|  |  |  |  |  | Q | P | | I² | |
| TLR4 mRNA |  |  |  |  |  | |  | |  |
| SF vs. HC | 2 | 169 | SMD(Ⅳ, REM) | -1.02(-2.17,0.13) | 12.0 | | 0.001 | | 91.7% |
| SF vs. SC | 2 | 174 | SMD(Ⅳ, FEM) | -0.90(-1.22,-0.59) | 0.8 | | 0.381 | | 0.0% |
| SC vs. HC | 2 | 169 | SMD(Ⅳ, REM) | -0.32(-1.26,0.62) | 8.9 | | 0.003 | | 88.7% |
| CCR3 |  |  |  |  |  | |  | |  |
| SF vs. HC | 1 | 70 | SMD(Ⅳ, FEM) | 4.29(3.42,5.16)* | - | | - | | - |
| SF vs. SC | 1 | 75 | SMD(Ⅳ, FEM) | 4.69(3.80,5.59) | - | | - | | - |
| HC vs. SC | 1 | 75 | SMD(Ⅳ, FEM) | 0.52(0.06,0.99) | - | | - | | - |
| CXCR4 |  |  |  |  |  | |  | |  |
| SF vs. HC | 1 | 70 | SMD(Ⅳ, FEM) | -0.05(-0.52,0.42) | - | | - | | - |
| SF vs. SC | 1 | 75 | SMD(Ⅳ, FEM) | 0.28(-0.18,0.74) | - | | - | | - |
| HC vs. SC | 1 | 75 | SMD(Ⅳ, FEM) | 0.35(-0.10,0.81) | - | | - | | - |

SF:Serofast Group;HC:Healthy Controls Group;SC:Serologically Cured Group;SP:Syphilis Patients

**P*<0.05

****Supplemental Material S7.**** Findings from Individual Studies on the Association Between miRNA and Syphilis Serofast

| Research Factor | ​Number of Studies | Study Population | Statistical Method | SMD(95%CI) |
| --- | --- | --- | --- | --- |
|  |  |  |  |  |
| miR-299-3P |  |  |  |  |
| SF vs. HC | 1 | 70 | SMD(Ⅳ, FEM) | 12.71(10.50,14.93) |
| SF vs. SP | 1 | 70 | SMD(Ⅳ, FEM) | 8.06(6.61,9.51) |
| HC vs. SP | 1 | 70 | SMD(Ⅳ, FEM) | -8.48(-10.00,-6.97) |
| miR-31 |  |  |  |  |
| SF vs. HC | 1 | 87 | SMD(Ⅳ, FEM) | -3.59(-4.29,-2.90) |
| SF vs. SC | 1 | 81 | SMD(Ⅳ, FEM) | -2.70(-3.31,-2.09) |
| HC vs. SC | 1 | 99 | SMD(Ⅳ, FEM) | 1.78(1.30,2.27) |
| miR-192 |  |  |  |  |
| SF vs. HC | 1 | 87 | SMD(Ⅳ, FEM) | -5.94(-6.93,-4.94) |
| SF vs. SC | 1 | 81 | SMD(Ⅳ, FEM) | -1.35(-1.83,-0.86) |
| HC vs. SC | 1 | 99 | SMD(Ⅳ, FEM) | 2.21(1.69,2.72) |
| miR-195 |  |  |  |  |
| SF vs. HC | 1 | 80 | SMD(Ⅳ, FEM) | 5.40(4.44,6.37)* |
| SF vs. SC | 1 | 80 | SMD(Ⅳ, FEM) | 1.63(1.12,2.14)* |
| HC vs. SC | 1 | 80 | SMD(Ⅳ, FEM) | -2.87(-3.50,-2.24) |
| miR-223 |  |  |  |  |
| SF vs. HC | 1 | 80 | SMD(Ⅳ, FEM) | 3.59(2.87,4.31)* |
| SF vs. SC | 1 | 80 | SMD(Ⅳ, FEM) | 0.94(0.47,1.40)* |
| HC vs. SC | 1 | 80 | SMD(Ⅳ, FEM) | -2.89(-3.53,-2.26) |
| miR-589 |  |  |  |  |
| SF vs. HC | 1 | 80 | SMD(Ⅳ, FEM) | 4.24(3.43,5.04)* |
| SF vs. SC | 1 | 80 | SMD(Ⅳ, FEM) | 0.95(0.49,1.41)* |
| HC vs. SC | 1 | 80 | SMD(Ⅳ, FEM) | -3.81(-4.55,-3.06) |

SF:Serofast Group;HC:Healthy Controls Group;SC:Serologically Cured Group;SP:Syphilis Patients

*P＜0.05
